# Inhibition of Ca^2+^/Calmodulin-dependent protein kinase II reverses oxaliplatin-induced mechanical allodynia in Rats

**DOI:** 10.1186/1744-8069-8-26

**Published:** 2012-04-17

**Authors:** Masafumi Shirahama, Soichiro Ushio, Nobuaki Egashira, Shota Yamamoto, Hikaru Sada, Ken Masuguchi, Takehiro Kawashiri, Ryozo Oishi

**Affiliations:** 1 Department of Pharmacy, Kyushu University Hospital, 3-1-1 Maidashi, Higashi-ku, Fukuoka 812-8582, Japan

## Abstract

**Background:**

Oxaliplatin is a key drug in the treatment of colorectal cancer, but it causes severe peripheral neuropathy. We previously reported that oxaliplatin (4 mg/kg, i.p., twice a week) induces mechanical allodynia in the late phase in rats, and that spinal NR2B-containig *N*-methyl-_D_-aspartate (NMDA) receptors are involved in the oxaliplatin-induced mechanical allodynia. In the present study, we investigated the involvement of Ca^2+^/calmodulin dependent protein kinase II (CaMKII), which is a major intracellular protein kinase and is activated by NMDA receptor-mediated Ca^2+ ^influx, in the oxaliplatin-induced mechanical allodynia in rats.

**Results:**

An increase of CaMKII phosphorylation was found in the spinal cord (L_4-6_) of oxaliplatin-treated rats. This increased CaMKII phosphorylation was reversed by intrathecal injection of a selective CaMKII inhibitor KN-93 (50 nmol, i.t.) and a selective NR2B antagonist Ro 25-6981 (300 nmol, i.t.). Moreover, acute administration of KN-93 (50 nmol, i.t.) strongly reversed the oxaliplatin-induced mechanical allodynia in von Frey test, while it did not affect the oxaliplatin-induced cold hyperalgesia in acetone test. Similarly, oral administration of trifluoperazine (0.1 and 0.3 mg/kg, p.o.), which is an antipsychotic drug and inhibits calmodulin, reduced both mechanical allodynia and increased CaMKII phosphorylation. On the other hand, trifluoperazine at the effective dose (0.3 mg/kg) had no effect on the paw withdrawal threshold in intact rats. In addition, trifluoperazine at the same dose did not affect the motor coordination in rota-rod test in intact and oxaliplatin-treated rats.

**Conclusions:**

These results suggest that CaMKII is involved in the oxaliplatin-induced mechanical allodynia, and trifluoperazine may be useful for the treatment of oxaliplatin-induced peripheral neuropathy in clinical setting.

## Background

Oxaliplatin, a platinum-based chemotherapeutic agent, has widely been used for colorectal cancer. However, oxaliplatin causes severe peripheral neuropathy. After multiple cycles, the patients develop a chronic neuropathy that is characterized by a sensory and motor dysfunction. This chronic neuropathy is a dose-limiting toxicity and a major clinical problem in oxaliplatin-based chemotherapy [1].

We previously reported that repeated administration of oxaliplatin induced cold hyperalgesia in the early phase and mechanical allodynia in the late phase in rats [2]. Recently, we reported that spinal NR2B-containing *N*-methyl-_D_-aspartate (NMDA) receptors are involved in the oxaliplatin-induced mechanical allodynia [3]. The NMDA receptor antagonists (MK-801 and memantine) and selective NR2B antagonists (Ro25-6981 and ifenprodil) reverse the oxaliplatin-induced mechanical allodynia. In addition, an expression of NR2B protein and mRNA in the rat spinal cord is increased by oxaliplatin on day 25 (late phase).

Activation of the NMDA receptors leads to an increase in Ca^2+ ^influx into the cytosol. This increased Ca^2+ ^influx initiates cascades of intracellular signaling events involving Ca^2+ ^and various protein kinases [4]. Ca^2+^/calmodulin dependent protein kinase II (CaMKII) is a major intracellular protein kinase and is activated by Ca^2+ ^signaling [5]. An increase in intracellular Ca^2+ ^initially activates calmodulin by binding to its Ca^2+^-binding sites, and this interaction induces a change in the conformation of calmodulin. CaMKII is then switched to an activated state by exposure to Ca^2+^/calmodulin. Several studies showed that an increase of CaMKII activation in the spinal cord is involved in persistent pain by nerve injury [6-9] and inflammation [10,11]. However, the role of CaMKII in the oxaliplatin-induced mechanical allodynia still remains unclear. In this study, we investigated the involvement of CaMKII in the oxaliplatin-induced mechanical allodynia, and explored novel useful therapeutic drugs for the oxaliplatin-induced neuropathy.

## Results

### Effects of KN-93 and KN-92 on Oxaliplatin-induced mechanical allodynia

**Figure 1:**
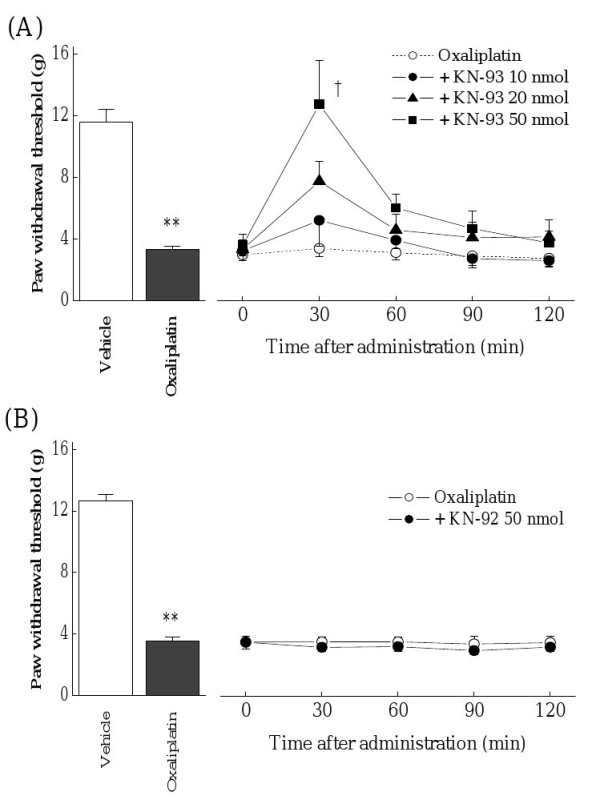
**Effects of KN-93 and KN-92 on oxaliplatin-induced mechanical allodynia in the von Frey test**. Rats were treated with oxaliplatin (4 mg/kg, i.p.) twice a week for 4 weeks (days 1, 2, 8, 9, 15, 16, 22 and 23). We confirmed the incidence of mechanical allodynia on day 24. We carried out the drug evaluation on the next day. KN-93 (10-50 nmol) or KN-92 (50 nmol) was administered intrathecally. The von Frey test was performed immediately before (0 min) and at 30, 60, 90 and 120 min after administration. KN-93 (50 nmol) significantly reversed oxaliplatin-induced mechanical allodynia (A). On the other hand, KN-92 (50 nmol) had no effect on the mechanical allodynia (B).Values are expressed as the mean ± SEM. of 5-8 animals. **p < 0.01 compared with vehicle (Student's *t*-test). †*p *< 0.05 compared with oxaliplatin alone (one-way ANOVA followed by Tukey-Kramer post-hoc test).

Oxaliplatin (4 mg/kg, i.p., twice a week for 4 weeks) significantly reduced the paw withdrawal thresholds compared with the vehicle in the von Frey test on day 24 (*p *< 0.01, Figure 1). Before administration of KN-93, each group had equivalent paw withdrawal thresholds. The selective CaMKII inhibitor KN-93 (50 nmol, i.t.) completely reversed the reduction of paw withdrawal thresholds by oxaliplatin at 30 min after the administration (*p *< 0.05, Figure 1A). This effect of KN-93 was disappeared within 120 min after the administration. On the other hand, treatment of KN-92 (50 nmol, i.t.), the negative control of KN-93, had no effect on the oxaliplatin-induced mechanical allodynia (Figure 1B).

### Effect of KN-93 on Oxaliplatin-induced cold hyperalgesia

**Figure 2:**
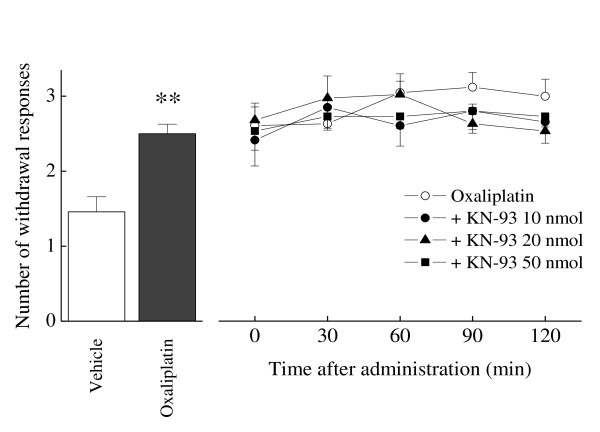
**Effect of KN-93 on oxaliplatin-induced cold hyperalgesia in the acetone test**. Rats were treated with oxaliplatin (4 mg/kg, i.p.) on days 1 and 2. We confirmed the incidence of cold hyperalgesia on day 5 and we carried out the drug evaluation on the same day. KN-93 (10-50 nmol) was administered intrathecally. The acetone test was performed immediately before (0 min) and at 30, 60, 90 and 120 min after administration. KN-93 had no effect on oxaliplatin-induced cold hyperalgesia. Values are expressed as the mean ± SEM. of 7-8 animals. **p <0.01 compared with vehicle (Student's *t*-test).

Oxaliplatin (4 mg/kg, i.p. on days 1 and 2) significantly increased the number of withdrawal responses to cold stimulation by acetone spray on day 5 (*p *< 0.01, Figure 2). Before administration of KN-93, each group had equivalent number of withdrawal responses. KN-93 (50 nmol, i.t.) did not affect the increase in withdrawal responses by oxaliplatin.

### Effects of KN-93 and Ro 25-6981 on Oxaliplatin-induced increase in spinal CaMKII phosphorylation

**Figure 3:**
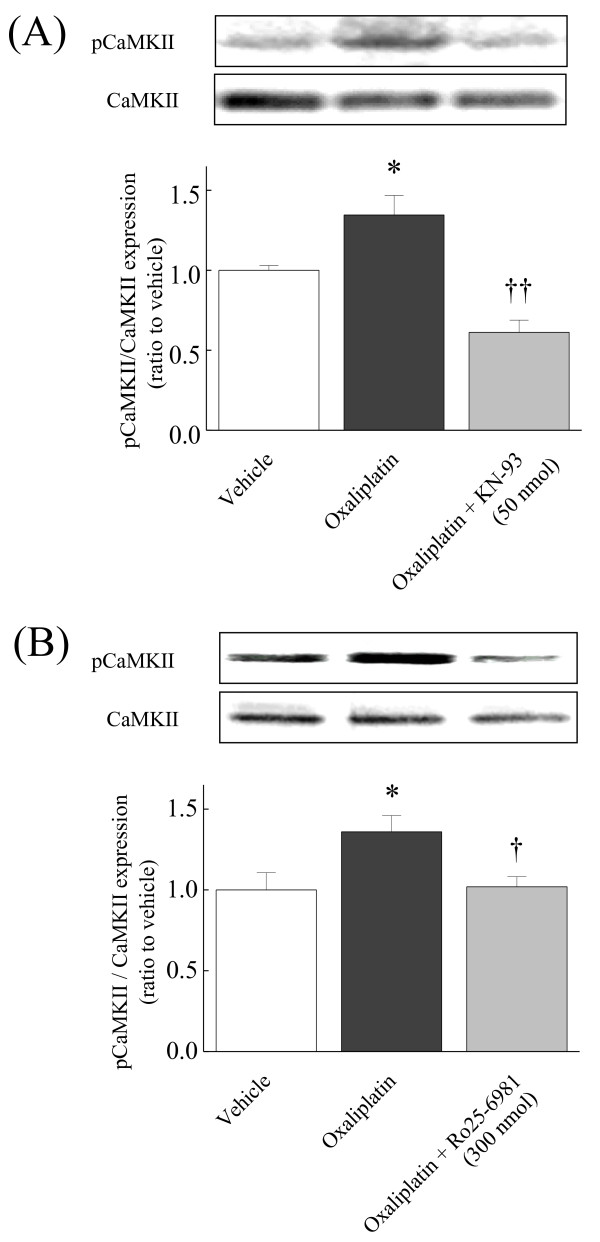
**Effects of KN-93 and Ro 25-6981 on oxaliplatin-induced increase in spinal CaMKII phosphorylation**. Rats were treated with oxaliplatin (4 mg/kg, i.p.) twice a week for 4 weeks (days 1, 2, 8, 9, 15, 16, 22 and 23). The lumbar sections (L_4-6_) of the spinal cord were quickly removed at 30 min after administration of KN-93 (50 nmol, i.t.) or Ro 25-6981 (300 nmol, i.t.) on day 25. CaMKII phosphorylation (pCaMKII) in the lumbar sections of the spinal cord was determined by Western blotting. An increase of pCaMKII was found in the spinal cord of oxaliplatin-treated rats. Acute treatment with KN-93 (A) and Ro 25-6981 (B) reduced oxaliplatin-induced increase in the spinal pCaMKII. Values are expressed as mean ± SEM. of 6-8 animals. *p < 0.05 compared with vehicle, †*p *< 0.05, ††*p *< 0.01 compared with oxaliplatin alone (one-way ANOVA followed by Tukey-Kramer post-hoc test).

To determine the effect of oxaliplatin on spinal CaMKII activity, we investigated the expression of CaMKII phosphorylation (pCaMKII). The pCaMKII in the spinal cord of oxaliplatin treated rats significantly increased compared with that of vehicle-treated rats on day 25 (*p *< 0.05, Figure 3). This increased pCaMKII was blocked by the selective CaMKII inhibitor KN-93 (50 nmol, i.t.) and the selective NR2B antagonist Ro 25-6981 (300 nmol, i.t.). (KN-93: *p *< 0.05; Ro 25-6981: *p *< 0.01, Figure 3).

### Effect of trifluoperazine on Oxaliplatin-induced mechanical allodynia and increase in CaMKII phosphorylation

**Figure 4:**
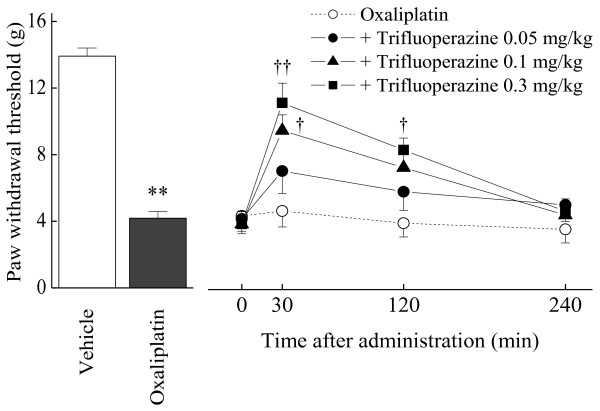
**Effect of trifluoperazine on oxaliplatin-induced mechanical allodynia in the von Frey test**. Rats were treated with oxaliplatin (4 mg/kg, i.p.) twice a week for 4 weeks (days 1, 2, 8, 9, 15, 16, 22 and 23). We confirmed the incidence of mechanical allodynia on day 24. We carried out the drug evaluation on the next day. Trifluoperazine (0.05-0.3 mg/kg) was administered orally. The von Frey test was performed immediately before (0 min) and at 30, 120 and 240 min after administration of trifluoperazine. Trifluoperazine (0.1 and 0.3 mg/kg) significantly reversed oxaliplatin-induced mechanical allodynia. Values are expressed as the mean ± SEM. of 8 animals. **p < 0.01 compared with vehicle, †*p *< 0.05, ††*p *< 0.01 compared with oxaliplatin alone (one-way ANOVA followed by Tukey-Kramer post-hoc test).

**Figure 5:**
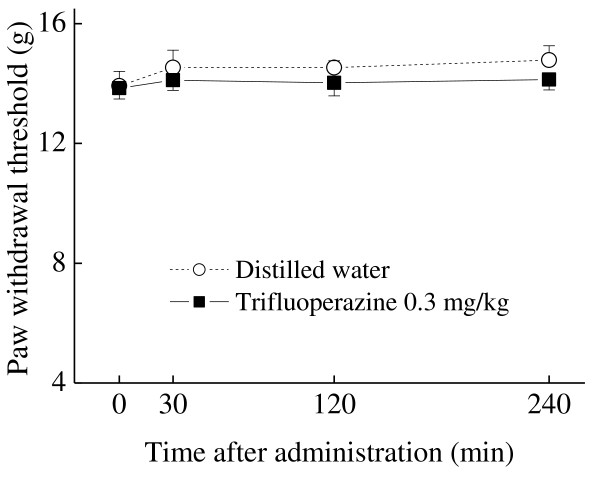
**Effect of trifluoperazine on mechanical nociceptive threshold in the von Frey test**. Trifluoperazine (0.3 mg/kg) was administered orally in intact rats. The von Frey test was performed immediately before (0 min) and at 30, 120 and 240 min after administration of trifluoperazine. Trifluoperazine did not affect mechanical nociceptive threshold in intact rats. Values are expressed as the mean ± SEM. of 8 animals. No statistical difference was identified (one-way ANOVA followed by Tukey-Kramer post-hoc test).

**Figure 6:**
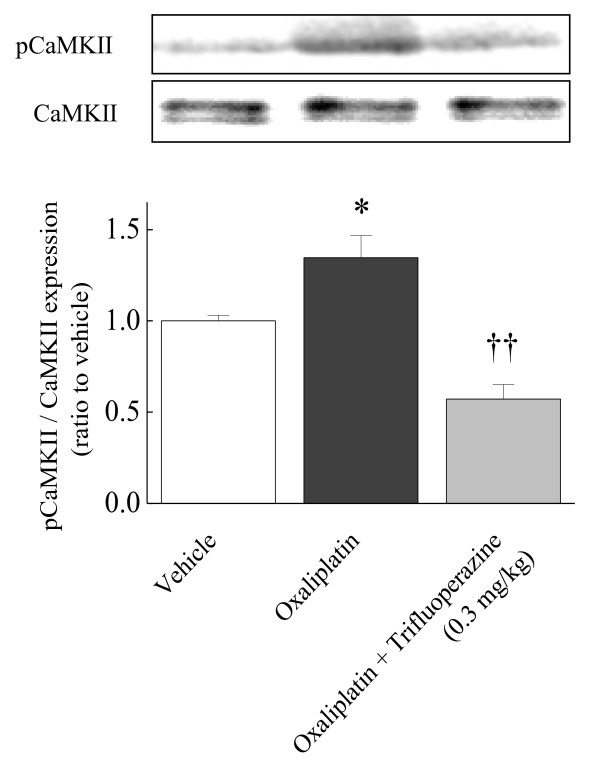
**Effect of trifluoperazine on oxaliplatin-induced increase of spinal CaMKII phosphorylation**. Rats were treated with oxaliplatin (4 mg/kg, i.p.) twice a week for 4 weeks (days 1, 2, 8, 9, 15, 16, 22 and 23). The lumbar sections (L_4-6_) of the spinal cord were quickly removed at 30 min after administration of trifluoperazine (0.3 mg/kg, p.o.) on day 25. CaMKII phosphorylation (pCaMKII) in the lumbar sections of the spinal cord was determined by Western blotting. An increase of pCaMKII was found in the spinal cord of oxaliplatin-treated rats. Acute treatment with trifluoperazine reduced oxaliplatin-induced increase in the spinal pCaMKII. Values are expressed as mean ± SEM. of 6-7 animals. *p < 0.05 compared with vehicle, ††*p *< 0.01 compared with oxaliplatin alone by Tukey-Kramer post-hoc test.

Since trifluoperazine, an antipsychotic drug, inhibits calmodulin required for CaMKII phosphorylation [12], we tested the effect of this compound on the oxaliplatin-induced mechanical allodynia. Trifluoperazine (0.1 and 0.3 mg/kg, p.o.) significantly reduced the oxaliplatin-induced mechanical allodynia at 30 min (0.1 mg/kg: *p *< 0.05; 0.3 mg/kg: *p *< 0.01) and 120 min (0.3 mg/kg: *p *< 0.05) after the administration (Figure 4). On the other hand, trifluoperazine at the effective dose (0.3 mg/kg, p.o.) had no effect on the paw withdrawal thresholds in intact rats (Figure 5). Trifluoperazine (0.3 mg/kg, p.o.) strongly reduced the oxaliplatin-induced increase in spinal pCaMKII (*p *< 0.01, Figure 6).

### Effect of trifluoperazine on motor coordination

**Figure 7:**
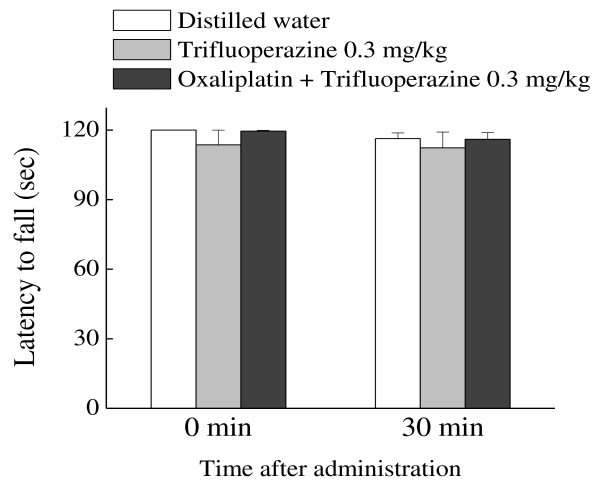
**Effect of trifluoperazine on motor coordination in the rota-rod test**. Trifluoperazine (0.3 mg/kg) was administered orally in intact and oxaliplatin-treated rats. The rota-rod test was performed immediately before (0 min) and at 30 min after administration of trifluoperazine. Trifluoperazine did not affect motor coordination in intact and oxaliplatin-treated rats. Values are expressed as the mean ± SEM. of 8-11 animals. No statistical difference was identified (one-way ANOVA followed by Tukey-Kramer post-hoc test).

We examined the influence of trifluoperazine on motor coordination in rota-rod test. At the time before (0 min) and 30 min after administration of trifluoperazine, there was no difference in the motor coordination among the groups-treated with distilled water, trifluoperazine 0.3 mg/kg or oxaliplatin + trifluoperazine 0.3 mg/kg (Figure 7).

## Discussion

In the present study, acute treatment of the CaMKII inhibitor KN-93 reversed the oxaliplatin-induced mechanical allodynia. KN-93 binds directly to calmodulin-binding site of CaMKII and inhibits the enzyme activity [13]. KN-93 also inhibits not only CaMKII but also L-type Ca^2+ ^channels [14], and therefore there is possibility that the effect of KN-93 on the mechanical allodynia depends on blockade of Ca^2+ ^channels. However, its structural analog KN-92, which does not inhibit CaMKII but blocks L-type Ca^2+ ^channels' current, did not reverse the oxaliplatin-induced mechanical allodynia. Moreover, the expression of pCaMKII significantly increased in the spinal cord of oxaliplatin-treated rats on day 25, and this increase of pCaMKII was blocked by KN-93. On the other hand, KN-93 had no effect on the oxaliplatin-induced cold hyperalgesia on day 5. These results indicate that the spinal CaMKII is involved in the oxaliplatin-induced mechanical allodynia but not cold hyperalgesia.

Recently, we reported that repeated administration of oxaliplatin increased the expression of NR2B protein and mRNA in the rat spinal cord on day 25 (late phase) but not on day 5 (early phase), and Ro 25-6981 reversed the oxaliplatin-induced mechanical allodynia [3], suggesting the involvement of NR2B-containing NMDA receptors. These NMDA receptors have been reported to contribute to development of spinal hyperexcitability and chronic pain [15-17]. Ca^2+ ^influx through activated NMDA receptors causes multiple intracellular changes including an activation of CaMKII [18,19], and the CaMKII co-localizes with NR2B in the nociceptive regions such as the superficial dorsal horn [20]. Furthermore, NR2B mutation, which causes a lower increase in intracellular Ca^2+ ^concentration by glutamate stimulation, leads to the reduction of spinal CaMKII phosphorylation in the neuropathic pain model [8,9]. In our present study, we found that the increase of pCaMKII in the spinal cord of oxaliplatin-treated rats was blocked by Ro 25-6981, the selective NR2B antagonist. Therefore, oxaliplatin may induce the CaMKII activation by increasing in the expression of NR2B-containing NMDA receptors. These findings indicate an important role of CaMKII as a downstream of NR2B-containing NMDA receptors in pain states.

In this study, we confirmed an inhibition of CaMKII phosphorylation by trifluoperazine, which inhibits calmodulin required for CaMKII activation [12]. Furthermore, we found that trifluoperazine dose-dependently reduced the oxaliplatin-induced mechanical allodynia. Previous studies showed that trifluoperazine reversed complete Freund's adjuvant-induced inflammatory pain and spinal nerve ligation-induced neuropathic pain in mice [7,11]. On the other hand, we observed that trifluoperazine at the effective dose (0.3 mg/kg) had no effect on the paw withdrawal thresholds in intact rats. In addition, trifluoperazine at the same dose did not affect the motor coordination in rota-rod test in intact and oxaliplatin-treated rats. Ye et al. [21] reported that trifluoperazine (0.125-0.5 mg/kg, i.p.) did not affect spontaneous locomotion in rats. Bhargava and Chandra [22] reported that ED50 (i.p.) of suppression of the conditioned response, an index of tranquilizing effect, is 0.58 mg/kg. Therefore, it is unlikely that the inhibitory effect of trifluoperazine on the oxaliplatin-induced pain behavior is due to its motor dysfunction or sedative effect. Taken together, the inhibitory effect of trifluoperazine on spinal CaMKII activity may be involved in the reduction of pain behavior, and low doses of trifluoperazine may be useful for the treatment of the oxaliplatin-induced neuropathy.

## Conclusions

Our results indicate that repeated administration of oxaliplatin increases spinal CaMKII activity. This increase of CaMKII activation was reversed by intrathecal injection of the selective CaMKII inhibitor and the selective NR2B antagonist. This CaMKII activation may contribute to the incidence of mechanical allodynia. Furthermore, the selective CaMKII inhibitor and the selective NR2B antagonist reduced the oxaliplatin-induced pain behavior. In addition, trifluoperazine reduced the oxaliplatin-induced mechanical allodynia and CaMKII activation. These results suggest that inhibition of CaMKII or NMDA-CaMKII pathway provides a novel therapeutic target for the treatment of the oxaliplatin-induced peripheral neuropathy.

## Methods

### Animals

Male Sprague-Dawley rats weighing 200-250 g (Kyudo Co., Saga, Japan) were used in the present study. Animals were housed in groups of four to five per cage, with lights on from 7:00 to 19:00 h. Animals had free access to food and water in their home cages. All experiments were approved by the Experimental Animal Care and Use Committee of Kyushu University according to the National Institutes of Health guidelines, and we followed International Association for the Study of Pain (IASP) Committee for Research and Ethical Issues guidelines for animal research [23].

### Drugs

Oxaliplatin (Elplat®) was obtained from Yakult Co., Ltd. (Tokyo, Japan). KN-93, Ro 25-6981 hydrochloride hydrate and trifluoperazine dihydrochloride were purchased from Sigma-Aldrich (Missouri, USA). KN-92 was purchased from Calbiochem (California, USA). Oxaliplatin was dissolved in 5% glucose solution. The vehicle-treated rats were injected with 5% glucose solution. KN-93, KN-92 and Ro 25-6981 were dissolved in 100% dimethyl sulfoxide (DMSO). Trifluoperazine was dissolved in distilled water. The doses of these drugs were chosen based on previous reports [2,3,7].

### Production of neuropathy

Mechanical allodynia and cold hyperalgesia were induced according to the method described previously [24]. Oxaliplatin (4 mg/kg) or vehicle (5% glucose solution) was administered i.p. twice a week for 4 weeks (on days 1, 2, 8, 9, 15, 16, 22 and 23). The volume of vehicle or drug solution injected was 1 mL/kg for all drugs.

### Behavioral studies

Behavioral test was performed blindly with respect to drug administration.

#### von Frey test for mechanical allodynia

The mechanical allodynia was assessed by von Frey test. Each rat was placed in a clear plastic box (20 × 17 × 13 cm) with a wire mesh floor and allowed to habituate for 30 min prior to testing. von Frey filaments (The Touch Test Sensory Evaluator Set; Linton Instrumentation, Norfolk, UK) ranging from 1- to 15-g bending force were applied to the midplantar skin of each hind paw six times, with each application held for 6 s. The paw withdrawal threshold was determined by a modified up-down method [25].

#### Acetone test for cold hyperalgesia

The cold hyperalgesia was assessed by acetone test. Each rat was placed in a clear plastic box (20 × 17 × 13 cm) with a wire mesh floor and allowed to habituate for 30 min prior to testing. Fifty microliters of acetone (Wako Pure Chemical Industries, Ltd., Osaka, Japan) was sprayed onto the plantar skin of each hind paw 3 times, and the number of withdrawal responses was counted for 40 s from the start of the acetone spray.

#### Rota-rod test for motor coordination

The rota-rod test was performed to investigate the change of motor coordination. Rats were placed on a rotating rod (Muromachi Kikai Co., Ltd., Tokyo, Japan) and the latency to falling was measured for up to 2 min according to the method described previously [26]. The test was performed three times, and the rotating speed was 10 rpm.

### Effects of KN-93, KN-92 and trifluoperazine on Oxaliplatin-induced mechanical allodynia

We confirmed the incidence of mechanical allodynia in the von Frey test on day 24. We carried out the drug evaluation on the next day. KN-93 (10-50 nmol) or KN-92 (50 nmol) was administered i.t. injection by direct lumbar puncture in a volume of 50 µL. The von Frey test was performed immediately before (0 min) and at 30, 60, 90 and 120 min after administration of the drugs. Trifluoperazine (0.05-0.3 mg/kg) was administered p.o. The von Frey test was performed immediately before (0 min) and at 30, 120 and 240 min after oral administration of trifluoperazine.

### Effect of KN-93 on Oxaliplatin-induced cold hyperalgesia

We confirmed the incidence of cold hyperalgesia in the acetone test on day 5. KN-93 (10-50 nmol) was administered i.t. injection by direct lumbar puncture in a volume of 50 µL. The acetone test was performed immediately before (0 min) and at 30, 60, 90 and 120 min after administration of the drug.

### Effect of trifluoperazine on mechanical nociceptive threshold

We investigated the effect of trifluoperazine on the mechanical nociceptive threshold in the von Frey test. Trifluoperazine (0.3 mg/kg) was administered p.o. in intact rats. The von Frey test was performed immediately before (0 min) and at 30, 120 and 240 min after oral administration of trifluoperazine.

### Effect of trifluoperazine on motor coordination

We investigated the effect of trifluoperazine on the motor coordination in the rota-rod test. Trifluoperazine (0.3 mg/kg) was administered p.o. in intact and oxaliplatin-treated rats. The rota-rod test was performed immediately before (0 min) and at 30 min after oral administration of trifluoperazine.

### Western blotting analysis

The lumbar sections (L_4-6_) of the spinal cord were quickly removed at 30 min after administration of KN-93 (50 nmol, i.t.), Ro 25-6981 (300 nmol, i.t.) or trifluoperazine (0.3 mg/kg, p.o.) on day 25. The tissues were homogenized in a solubilization buffer containing 20 mM Tris-HCl (pH 7.4, 2 mM EDTA, 0.5 mM EGTA, 10 mM NaF, 1 mM Na_3_VO_4_, 1 mM PMSF, 0.32 M Sucrose, 2 mg/ml aprotinine, 2 mg/ml leupeptin), and the homogenates were subjected to 12.5% SDS-PAGE, and proteins were transferred electrophoretically to PVDF membranes. The membranes were blocked in Tris-buffered saline Tween-20 (TBST) containing 5% BSA (Sigma-Aldrich) for an additional 1 h at room temperature with agitation. The membrane was incubated overnight at 4°C with mouse polyclonal anti-CaMKIIα antibody or rabbit polyclonal anti-(Thr286)pCaMKII (1:5000; Santa Cruz Biotechnology, California, USA) and then incubated for 1 h with corresponding horseradish peroxidase conjugate secondary antibodies (1:5000; Jackson Immuno Research Laboratories, Inc., PA, USA). The immunoreactivity was detected using Enhanced Chemiluminescence (Perkin Elmer, Massachusetts, USA). Ratios of the optical densities of pCaMKII to those of CaMKII were calculated for each sample.

### Data analysis

Values were expressed as the means ± SEM. The values were analyzed by the Student's *t*-test or one-way analysis of variance (ANOVA) followed by the Tukey-Kramer post-hoc test (StatView; Abacus Concepts, Berkely, CA, USA) to determine differences among the groups. A *p *value of less than 0.05 is considered as statistically significant.

## Competing interests

The authors declare that they have no competing interests.

## Authors' contributions

NE and TK are responsible for experimental design. MS, SU and HS are responsible for performance of behavioral tests. MS, SU, SY, HS and KM are responsible for performance of Western blotting. NE, MS and RO are responsible for writing the manuscript. All authors read and approved the final manuscript.
